# Current Approaches to Prevent or Reverse Microbiome Dysbiosis in Chronic Inflammatory Rheumatic Diseases

**DOI:** 10.31138/mjr.240224.cap

**Published:** 2024-06-30

**Authors:** Jan René Nkeck, Ange Larissa Tchuisseu-Kwangoua, Adeline Pelda, Wilson Chia Tamko, Saquinatou Hamadjoda, Doris Bibi Essama, Baudelaire Fojo, Moustapha Niasse, Saïdou Diallo, Madeleine Ngandeu-Singwé

**Affiliations:** 1 Yaoundé Rheumatology Research Team, Yaoundé, Cameroon,; 2 Department of Internal Medicine and Specialties, Faculty of Medicine and Biomedical Sciences, University of Yaoundé I, Yaoundé, Cameroon,; 3 Nuffield Department of Population Health, University of Oxford, Oxford, United Kingdom,; 4 Rheumatology Unit, Yaoundé Central Hospital, Yaoundé, Cameroon,; 5 Department of Rheumatology, Dantec Teaching Hospital, Cheikh Anta Diop University, Dakar, Senegal

**Keywords:** dysbiosis, prevention, treatment, chronic inflammatory rheumatic diseases, microbiome, sub-Saharan Africa

## Abstract

Advances in knowledge of the microbiome and its relationship with the immune system have led to a better understanding of the pathogenesis of chronic inflammatory rheumatic diseases (CIRD). Indeed, the microbiome dysbiosis now occupies a particular place with implications for the determinism and clinical expression of CIRD, as well as the therapeutic response of affected patients. Several approaches exist to limit the impact of the microbiome during CIRD. This review aimed to present current strategies to prevent or reverse microbiome dysbiosis based on existing knowledge, in order to provide practical information to healthcare professionals treating patients suffering from CIRD.

## BACKGROUND

Chronic inflammatory rheumatic diseases of adults represent a heterogeneous group of conditions responsible for persistent inflammation within the anatomical structures that build up the musculoskeletal system. They can be autoimmune, dominated by systemic lupus, rheumatoid arthritis, systemic scleroderma, idiopathic inflammatory myopathies and Sjögren’s syndrome, or autoinflammatory, dominated by spondyloarthritis, certain vasculitis, sarcoidosis, and crystal induced arthropathies.^1^ Even though many of these conditions are still considered rare in certain populations, they represent a real public health problem and occupy a key place in rheumatology consultations. The determinants of CIRD are mainly genetic, immunological, epigenetic, and environmental.^2^ A deep insight into these factors is critical not only for refining diagnostic and therapeutic strategies, but also for shaping prevention methods tailored to target populations. Over the last few decades, understanding the involvement of the microbiome has revolutionised our knowledge of the pathogenesis of inflammatory diseases in rheumatology.^3^ Dysbiosis represents an imbalance in the constitution of the microbiome, which used to have a symbiotic relationship with the immune system. There is a great deal of evidence to suggest that this imbalance may contribute to the development of CIRD, and may also affect their activity and the therapeutic response of patients.^4^ Understanding dysbiosis holds significant promise for advancing our knowledge of disease mechanisms and exploring innovative therapeutic avenues in the realm of rheumatology. Therefore, it is crucial to highlight the potential applications of what is already known, particularly for the prevention and management of microbiome dysbiosis. The aim of this review is to address this issue and provide sufficient practical information to clinicians caring for patients with CIRD.

## METHODS

This is a narrative review of the existing literature published on PubMed/Medline up to 31 January 2024 without time or language restriction. We used the following terms for research: ”microbiome”, “microbiota”, “dysbiosis”, “microbial community”, “chronic, inflammatory rheumatic diseases”, “systemic lupus erythematosus”, “rheumatoid arthritis”, “systemic sclerosis”, “Sjögren syndrome”, “Idiopathic inflammatory myopathy”, “spondylarthritis”, “reactive arthritis”, “gout”, “sarcoidosis”, “vasculitis”. Articles that discussed the preventive and curative strategies for microbiome dysbiosis related on CIRD were retained after screening and examination by the authors. We did not carry out a meta-analysis as this was a descriptive review that did not require statistical factor estimation. The figures were designed using the licensed online version of the BioRender software.

## UNDERSTANDING THE MICROBIOME

### Definition and history

In the seventeenth century, Antony van Leeuwenhoek, often hailed as the father of microbiology, made ground-breaking observations by describing millions of micro-organisms, referred to as “animalcules,” residing within the plaque of his gums.^5^ This early exploration into the microbial world laid the foundation for understanding the complex human microbiome. The microbiome is constituted by the vast array of microorganisms (microbiota - such as fungi, bacteria and viruses) inhabiting various human body cavities and surfaces, encompassing both its genetic and enzymatic composition.^6^ The presence of this collective microbial community defines our microbiome. Today, our knowledge of the microbiome has significantly expanded, thanks in part to the Human Microbiome Project, which aimed to characterise microbial communities throughout the human body.^7^

### Types of microbiome

Human microbiome consists of a core microbiome and a variable microbiome. The core microbiome is common to all the individuals, whereas variable microbiome is unique to individuals and take into account the genetic and ecological difference (microbial count and diversity) within the microbiome.^8^ We can classify microbiomes according to their location on the human body.^9^ A distinction is made between: the microbiome of the skin, the oral cavity, the gastrointestinal tract (gut), the respiratory tract, and the urogenital tract. There are other specific types of micro-biome depending on their location, including the blood vessel microbiome.

### Microbiome and host symbiosis

To survive within the human body, the microbiome maintains a symbiotic relationship with the host. Understanding the microbiome’s role has evolved over time, emphasising its pivotal role in shaping key aspects of human biology. The benefits of this symbiosis for the human body can be both metabolic and immunological. In terms of immunity, colonisation by the microbiome discourages the presence of harmful germs; it plays a part in modulating both the innate and adaptive immune responses.^10^ It promotes the reduction of pro-inflammatory cytokines and the production of anti-inflammatory cytokines. On a metabolic level, it is involved in the homeostasis of epithelia, particularly the intestinal epithelium (bile acid cycle), digestion and production of short-chain fatty acids, and vitamin synthesis.^11^ In parallel, the immune system has evolved to establish a mutually beneficial relationship with the constantly changing microbial community.^12^ Antigen presenting cells protect the body against infection without modifying the immunological tolerance to normal microbiota. For example, dendritic cells (DCs) of Peyer’s patches produce a great number of interleukin-10, compared with splenic DCs activated under similar conditions.^13^ Similarly, gut macrophages are proximal to the intestinal microbiota, and they develop a unique phenotype, so called “inflammation anergy,” referring to the non-inflammatory profile of intestinal macrophages when they encounter microbial stimuli in homeostatic conditions.^14^ In addition, the microbiome synthesises short-chain fatty acids preferentially from the fermentation of fibres, which help to reduce inflammation.^15^

### Microbiome dysbiosis

The microbiome varies within the same individual, influenced by genetic, lifestyle and environmental factors. The main variables influencing the composition of the microbiome in newborns and children can be: mode of delivery and breastfeeding, hygiene, exposure to environmental agents, genetic background; and in adults: diet, antimicrobials, alcohol consumption, smoking, age, sex, stress, and obesity.^16^ The symbiosis between the microbiome and the host implies an eubiosis within the microbiome, i.e. a balance within the microbiome where beneficial germs tolerated by the immune system predominate; these control potentially harmful germs. What’s more, there must be a physiological functioning of the immune system that is tolerant of and cooperative with the beneficial germs.^11^ An imbalance in the constitution and function of the microbiome where germs not tolerated by the immune system predominate and/or are not controlled may be transitory, leading to a transient low-grade inflammation. A sustained imbalance is referred to dysbiosis and will cause sustained inflammation which may be low or high grade, local, or systemic.^8^ From the dysbiosis, there will be a translocation of immunogenic bacterial products like lipopolysaccharides, leading to the activation of innate and adaptive immunity resulting in inflammation, activation of Toll like receptor 4 and several signalling pathways (JAK/STAT, NF/kB) leading to the production of inflammatory cytokines (TNF alpha, interleukin 1, MCP1, interleukin 12).^17^

## MICROBIOME AND CIRD

**Figure 1:**
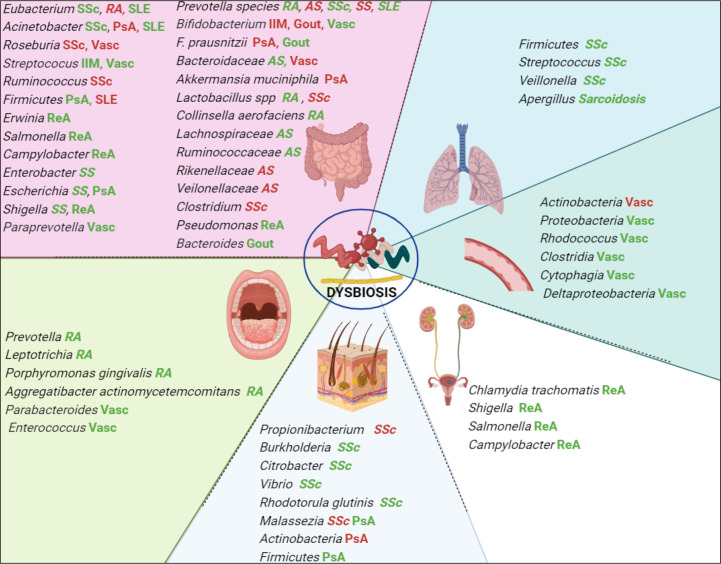
Chronic inflammatory rheumatic diseases and mostly associated dysbiosis. AS: ankylosing spondylarthritis; IMM: idiopathic inflammatory myositis; PsA: psoriatic arthritis; RA: rheumatoid arthritis; ReA: reactive arthritis; SLE: systemic lupus erythematosus; SS: Sjögren’s syndrome; SSc: systemic sclerosis; Vasc: vasculitis.

Numerous studies have demonstrated the involvement of dysbiosis in the pathogenesis of CIRD. Dysbiosis may be involved not only in the genesis of autoimmunity and autoinflammation, but may also have an impact on CIRD activity and therapeutic response. **Figure 1** shows the main dysbiosis observed in CIRD. **Table 1** shows the main microorganisms observed associated with CIRD.

**Table 1.** Different chronic inflammatory rheumatic diseases and associated dysbiosis.

**Rheumatic disease**	**Site**	**Microbiomes components involved**	**References**
Systemic sclerosis	Gut	Bacteroides, Faecalibacterium, Clostridium, Fusobacterium, Prevotella, Lactobacillus	[18,19]
	Skin	Rhodotorula glutinis, Propionibacterium) Malassezia, Burkholderia, Citrobacter, Vibrio	[18,20,21]
	Lung	Firmicutes, Streptococcus, and Veillonella	[22]
Psoriasis and Psoriatic arthritis	Gut	Escherichia coli, Firmicutes, Actinobacteria, Faecalibacterium prausnitzii and Akkermansia muciniphila	[23–25]
	Skin	Malassezia, Staphylococcus, Propionibacterium, Actinobacteria, Corynebacterium spp	[26–28]
Rheumatoid arthritis	Oral	Prevotella, Leptotrichia, lactobacillus, Porphyromonas gingivalis, Aggregatibacter actinomycetemcomitans	[29–34]
	Gut	Prevotella species, lactobacillus spp - Collinsella aerofacien	[29,35–37]
Reactive arthritis	Gut	Erwinia and Pseudomonas, Shigella, Salmonella, and Campylobacter	[38,39]
	Urogenital	Chlamydia trachomatis, Shigella, Salmonella, Campylobacter	[38–40]
Sjögren syndrome	Gut	Clostridium, Enterobacter, Escherichia/Shigella, Pseudomonas	[41,42]
Spondylarthritis	Gut	Lachnospiraceae, Ruminococcaceae, Rikenellaceae, Porphyromonadaceae, Bacteroidaceae, Veilonellaceae, Prevotellaceae	[43,44]
Systemic lupus erythematosus	Gut	*Firmicutes, Bacteroidetes, Rhodococcus, Klebsiella, Prevotella, Eubacterium*	[33,45–47]
Idiopathic inflammatory myopathies	Gut	*Streptococcus, Lachnoclostridium, Tyzzerella* 3, *Tyzzerella 4, Bifidobacterium, Christensenellaceae R-7 group, Anaerostipes*	[48]
Gout	GUT	*Bacteroides, Prevotella, Bifidobacterium,*	[49–52]
Giant cell arteritis	Vascular	*Actinobacteria, Proteobacteria, Rhodococcus*	[53–56]
Small vessel vasculitis	Oral	*Parabacteroides, Enterococcus*	[54,56–58]
Medium vessel vasculitis	Gut	*Streptococcus, Bacteroidetes, Dorea*	[54,56 ,59–62]
Takayasu arteritis	Blood and blood vessel microbiome	*Clostridia, Cytophagia, Deltaproteobacteria*	[55,56]
Behçet’s disease	Gut	*Bilophila, Alistipes, and Paraprevotella*	[63]
Sarcoidosis	Respiratory	*Apergillus*	[64]

### Microbiome, autoinflammation and autoimmunity

The microbiome has been associated with a number of autoimmune and autoinflammatory diseases, both in animal models and in humans: rheumatoid arthritis, systemic lupus, Sjögren’s syndrome, scleroderma, antiphospholipid syndrome, ankylosing spondylitis, psoriatic arthritis, chronic inflammatory bowel diseases, etc.^65–67^ However, this association is complex, involving several mechanisms depending on the microbiome and the pathology. Nevertheless, the microbiome alone cannot explain all the process. For example, data on the gut microbiome have shown that in the eubiosis state, the metabolites produced by the microbiome (short-chain fatty acids, equol) are beneficial and maintain the anergy of macrophages and dendritic cells, and a balance between regulatory T lymphocytes, which are anti-inflammatory, producing interleukin 10 and 35 and TGF beta, and helper T lymphocytes 17, which are pro-inflammatory.^65,66^ Dysbiosis will lead to a loss of tolerance of the microbiome by the immune system, which will continually produce metabolites such as lipopolysaccharides and petidoglycans, coupled with a loss of tight junctions in the mucosa, which increases permeability and therefore microbial translocation into the systemic circulation.^68^ These phenomena can activate innate and adaptive immunity, via macrophages and dendritic cells, and lead to an imbalance in the profile of T helper 17 lymphocytes with the production of proinflammatory mediators such as TNF alpha, interleukins (1 beta, 6, 12, 17, 18, and 33), and numerous granulocyte growth factors. They also trigger the generation of autoreactive T and B lymphocytes, either by molecular mimicry, epitope propagation, or under the influence of metabolites from dysbiosis.^65,66^ It should also be noted that certain authors have shown that there is a particular interaction between HLA B27 and dysbiosis which favours the development of spondyloarthritis in animal models.^69^

### Microbiome, chronic inflammatory rheumatic disease activity, and therapeutic response

In addition to immunopathogenesis, the influence of the microbiome on the activity of chronic inflammatory rheumatism has been demonstrated. Dysbiosis maintains chronic inflammation and therefore regular activation of the immune system. It amplifies autoimmunity and autoinflammation via post-translational modifications of autoantigens, neutrophil death and cross-reactions.^70^ Certain bacterial taxa, such as *Prevotella copri* and *Collinsella spp*., have been associated with disease activity in rheumatoid arthritis (RA).^71^ Conti et al. revealed commensals of *Staphylococcus aureus* in the nasal mucosa of SLE (systemic lupus erythematosus) patients with influence on disease activity.^72^ Corrêa et al. revealed in SLE patients that *Fretibacterium*, *Prevotella nigrescens*, and *Selenomonas* spp. was a culprit in the release of interleukin- (IL6, IL17, and IL33).^73^ Andréasson et al. on the other hand, found 75.5% of dysbiosis in severe cases of gastrointestinal symptoms in SSc (systemic sclerosis).^74^ Dysbiosisis with *Bifidobacterium species* in Sjögren’s syndrome interferes with immune response to microbes and may have an influence on diseases activity.^75^ Systemic inflammation has been associated to gut dysfunction in ankylosing spondylitis (AS) and psoriatic arthritis.^76,77^ With knowledge on the impact of the microbiome on disease activity, its undeniable that its dysfunction will gain influence over the severity, progression, and the development of complications of CIRD. In RA, specific gut bacterial taxa such as *Prevotella copri* have been linked to more severe radiographic joint damage and functional disability.^71,78^

The composition of the gut microbiota has been suggested to impact the response to therapeutic interventions in CIRD. It seems to have a relationship between gut microbial composition and the efficacy of disease-modifying ant rheumatic drugs (DMARDs) in RA, especially for certain bacterial taxa (*Faecali bacterium prausnitzii* and *Bacteroides fragilis*.^79^ Additionally, gut microbial metabolites can influence the Pharmacodynamics and pharmacokinetics of DMARDs, like methotrexate and sulfasalazine, influencing therapeutic options.^79^ Biological DMARDs directly or indirectly interferes with inflammatory cytokines production which roles are determinant on microbiome dysbiosis effect on the activity of CIRD.^80^ Some authors have also shown that targeting microbiome dysbiosis can be an adjuvant treatment in CIRDs such as RA.^81^

## PREVENT OR REVERSE MICROBIOME DYSBIOSIS

**Figure 2:**
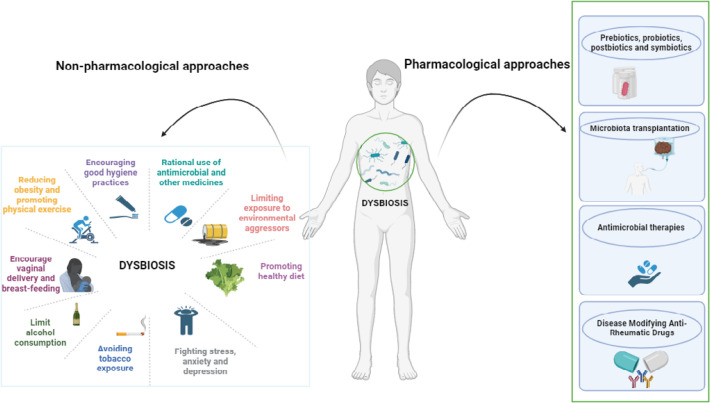
Strategies for preventing and restoring dysbiosis in the microbiome.

Preserving the microbiome or reversing dysbiosis and restoring balance within it is a current concern. Several actions are possible based on studies of the microbiome. They can be applied to all microbiomes, or be specific to certain microbiomes or specific age groups. They can be divided into pharmacological and non-pharmacological approaches. **Figure 2** summarises the various actions that can be taken to prevent or restore dysbiosis.

## NON-PHARMACOLOGICAL APPROACHES

Non-pharmacological approaches are the easiest to implement. They can be planned at individual, community, national and even international level. They are not specific to chronic inflammatory rheumatic diseases, and can be used in a wide range of conditions, including cardiovascular diseases. They include: encouraging good hygiene practices, rational use of antimicrobials and other medicines, limiting exposure to environmental aggressors, promoting healthy diet, reducing obesity, promoting physical exercise, limit exposure to alcohol and tobacco, fighting stress, anxiety, and depression.

### Encouraging good hygiene practices

Hygiene habits can affect the composition of the skin, respiratory, oral, intestinal and vaginal microbiomes. Hygiene habits can affect the composition of the micro-biome directly, for example the skin microbiome affected by the type of soap used for the body or the vaginal microbiome. In addition, a lack of hygiene can encourage infections by germs which in turn disrupt the balance of the microbiome.^82^ Examples include respiratory infections caused by poor environmental hygiene, digestive infections caused by poor food hygiene, and skin dysbiosis caused by colonisation by Staphylococcus aureus.^83–85^ Traditional body hygiene measures have been shown to reduce the colonisation of pathogenic germs and therefore dysbiosis. For example, the use of traditional oral hygiene methods reduces the microbial load in the oral cavity.^86^ It is therefore essential to promote good hygiene practices including personal hygiene (skin, oral, vaginal), food hygiene and environmental hygiene.

### Rational use of antimicrobials and other medicines

Antimicrobial consumption alters the balance of the microbiome and can lead to dysbiosis. Antimicrobials may be used directly to treat an infectious disease or absorbed indirectly through feed from animal products exposed to antibiotics.^87,88^ This is particularly described in the literature for the gut microbiome. It is of paramount importance to reduce the harmful effects of antimicrobial use on the microbiome. This needs to be part of a national and international strategy to promote the rational use of antimicrobials in hospital and out-of-hospital settings, as well as part of a “one health” strategy program to involve stakeholders in the animal husbandry and veterinary sectors.^89,90^ A number of non-antimicrobial drugs can affect the composition of the gut microbiome. Maier et al. evaluated the impact of non-antibiotic drugs such as proton pump inhibitors, metformin, non-steroidal anti-inflammatory drugs, statins, opioids, calcium channel blockers, thyroid hormones, antimetabolites, and antipsychotics, and found that each of these substances altered the functioning of at least one microbial strain at intestinal level.^91^ Although little data is currently available on this subject, the use of these treatments needs to remain within the indications in order to reduce their effect on dysbiosis.

### Limiting exposure to environmental aggressors

Exposure to environmental toxins is a major determinant of dysbiosis. These include air pollution, which can have an impact on the respiratory, oral, gut and even skin microbiomes.^92,93^ They can also include a wide range of environmental chemicals, such as heavy metals, pesticides, microplastics, nanoparticles, and even endocrine disruptors.^94,95^ On the skin, the action of ultraviolet light has mainly been reported, as it can alter the balance of the skin microbiome.^96^ They can act directly on the microbiome or cause genetic or epigenetic alterations which in turn promote an imbalance in the microbiome.^97^

In practice, this means limiting exposure to pollutants in the air, water, and food. This involves environmental studies to estimate their concentrations in the environment, a commitment to improve the environment, avoiding the use of pollutants and promoting the use of sun creams to protect against ultraviolet radiation in the event of exposure. An important point is the particular role of climate, which is rather complex and remains under-appreciated to this day; it involves, in a combined way, the effect on the human organism of seasonal ambient temperatures in an environmental context that may itself be subject to pollution, but also food (plant and animal) that has matured in these specific conditions and that will be consumed by the human organism. It has been shown that climatic variations, particularly in temperature, can have an influence on microbial diversity, particularly in the intestinal microbiome.^98^ However, the effects of climatic interventions on dysbiosis have yet to be assessed.

### Promoting healthy diet

The composition of the gut microbiome is modulated by diet independently of weight and adipose tissue activity. It has been shown that a high-fibre diet, adequate water intake, avoidance of saturated fatty acids, rapid sugars, protein rich diet and processed foods has a protective effect against intestinal dysbiosis. The germs in the microbiome feed primarily on carbohydrates produced by the fermentation of fibres and undigested polysaccharides, which are mainly found in plants and resistant starch-based foods. A diet low in these elements will reduce the production of short-chain fatty acids and encourage local inflammation.^15^ Several types of diet have been evaluated for their effect on the microbiome. The Western diet, which is described as rich in animal proteins, saturated fatty acids and low in dietary fibre, will favour a dysbiosis, particularly reducing the diversity of *Bifidobacterium* and *Eubacterium* and favouring the multiplication of *Enterobacteria* and *Bacteroides*, which will produce lipopolysaccharides that will activate the inflammatory response.^99,100^ The Mediterranean diet, rich in mono- and polyunsaturated fatty acids, indigestible vegetable fibre, carbohydrates with a low glycaemic index, polyphenols, antioxidants and micronutrients. It has been associated with an increase in germs such as *Prevotella*, *Lactobacillus,* and *Bifidobacterium,* and a decrease in *Clostridium*, which is beneficial for the balance of the intestinal microbiome.^101,102^ Vegetarian and vegan diets, which are rich in fermentable fibre and plant-based foods, will stimulate an increase in *Bacteriodes* and *Bifidobacterium sp*, which will lead to the production of short-chain fatty acids with an anti-inflammatory effect within the microbiome, influencing intestinal pH and bacterial colonisation.^103^ The gluten-free diet appears to encourage dysbiosis by creating a proliferation of *Enterobacteria*, *E. coli*, *Clostridiaceae*, *Victivallaceae*, etc.^104^ Intermittent fasting is reported to be beneficial, with a proliferation of germs such as *Ruminococcaceae* and *Roseburia* resulting in a decrease in lipopolysacvharides and an increase in short-chain fatty acids, thus reducing inflammation and dysbiosis.^105^ The Atlantic diet, which is rich in vitamin B, omega-3 fatty acids, and iodine, has been reported to be beneficial in regions where it is consumed.^106^ In sub-Saharan Africa, a number of foods and diets can promote the host’s immune defences, as well as the immunotolerance and balance of the microbiome. The effects of some of these so-called indigenous diets have been evaluated by several authors, in particular plant foods such as tapioca, millet, soya, and many others consumed by people in several African countries, with the potential of reversing dysbiosis within the intestinal microbiome by acting on germs such as *Bifidobacterium*, *Prevotella*, *Clostridium,* and *Shigella.*^107,108^ This sub-Saharan diet, rich in fibre and polysaccharides, is a potential therapeutic and probiotic food for the intestinal microbiome dysbiosis and need to be more evaluated and therefore promoted.^109^

### Reducing obesity

There is a complex bidirectional relationship between obesity and the microbiome, involving genetics and the environment.^110^ On the one hand, the constitution of the microbiome is particular in obese people, and the genetics, diet and physical inactivity associated with obesity maintain dysbiosis.^111^ In addition, dysbiosis promotes obesity by several mechanisms: biosynthesis of short-chain fatty acids, increased intestinal permeability to lipopolysaccharides promoting insulin resistance and inflammation, and increased intestinal endocannabinoid activity.^112,113^ The fight against obesity is therefore essential in order to act on dysbiosis. It is well codified and involves preventive measures such as information and education, promotion of a healthy diet and regular physical activity; screening measures and therapeutic measures which may be non-pharmacological (weight loss diets, physical activity), medicinal and/or surgical.^114^ Among weight-loss drug strategies, there is particular interest in Glucagon like peptide 1 (GLP-1) and Glucagon like 1 receptor (GLP-1R) agonist drugs (Tirzepatide, semaglutide, Liraglutide), which have been associated with improved dysbiosis and balance of the gut-brain axis.^115,116^ Other antidiabetic agents not used for weight loss, such as metformin, sitagliptin and acarbose, may among other effects reduce the synthesis of short-chain fatty acids in the gut and help restore the balance of the microbiome.^116^

### Promoting physical exercise

Physical exercise has been reported to have a protective effect on the microbiome, particularly in studies on chronic inflammatory bowel disease. It has been shown to induce the production of anti-inflammatory cytokines such as myokines, irisin, interleukin 15, and myonectin, coupled with a reduction in the levels of NLR-3 and caspase 1, which are involved in the inflammasome in obese people and children. Although it is important to promote regular physical exercise, there are as yet no specific recommendations in terms of duration and type of exercise.^117^

### Limit alcohol consumption

Alcohol consumption is a determining factor in the balance of the microbiome, particularly the oral and intestinal microbiome.^118^ The dysbiosis observed in these microbiomes has been linked to the quantity of alcohol consumed.^119^ Alcohol consumption leads to a reduction in certain commensal bacteria in the digestive tract, in particular *Lactobacilli* and *Enterococci spp*, and an increase in certain pathogenic bacteria such as *Klebsiella*, *Shigella*, Salmonella, and Escherichia coli.^120^ Alcohol also disrupts the intestinal barrier by causing mucosal permeability, which favours the passage of immunogenic bacterial components into the circulation and hence the inflammatory response.^121^ The dose at which alcohol affects the microbiome is not yet known. All this reinforces the importance of limiting alcohol consumption if we want to prevent its effects on the microbiome. This requires codified strategies to support drinkers.^122^

### Avoiding tobacco exposure

Exposure to tobacco, regardless of age, dose or type, is associated with significant changes in the microbiome.^123^ Numerous studies have demonstrated this in animal models and in humans.^124–126^ These changes apply to both the oral and respiratory microbiome and the intestinal microbiome, and may be due to nicotine and other components of cigarettes, in particular aldehydes, polycyclic aromatic hydrocarbons, heavy metals, toxic gases, and volatile organic substances.^127^ Limiting exposure to tobacco is a decisive factor in preventing and restoring its effects on the microbiome. Individual and community action (avoidance, cessation, and support) must be combined with national and international action on tobacco regulations.^128^ Among smoking-cessation strategies, frequently used e-cigarettes may represent a potential danger to the microbiome; particularly, the gut microbiome. It may promote inflammation and lesions in exposed mucosa, leading to barrier breakdown due to proinflammatory cytokines and infection, evolving into chronic inflammation within a dysbiotic gut microbiome.^129^ Even if these observations are still mostly based on animal models rather than humans, it is advisable to discourage this practice in order to preserve the balance of the microbiome during smoking cessation.

### Fighting stress, anxiety and depression

Stress, anxiety, and depression are also associated with dysbiosis, particularly intestinal dysbiosis, which is part of the brain-gut axis.^130^ Stress affects eating habits, and stress hormones interact with elements of the micro-biome and the immune system.^131^ The relationship is also bidirectional. The microbiome can in turn produce metabolites, toxins and even neurohormones that can induce behavioural changes.^132^ In order to preserve the microbiome, strategies to combat stress must be instituted at both individual and community level. Anxiety and depression must be identified and effectively managed.

### Prevent dysbiosis in newborns

Neonates inherit a large part of their microbiome through vaginal passage and maternal breastfeeding. To prevent dysbiosis, it is important to encourage vaginal delivery except in contraindicated cases, and to promote breast-feeding.^16^

## PHARMACOLOGICAL APPROACHES

Several therapeutic measures have an effect on the microbiome. They essentially correct dysbiosis. Most of them are still experimental and costly. It is therefore essential to initially demonstrate dysbiosis before undertaking certain techniques. Pharmacological approaches include prebiotics, probiotics, postbiotics, symbiotics, microbiota transplantation, and antimicrobial therapies. It is also important to consider the effects of DMARDs (Disease-Modifying Antirheumatic Drugs) on the micro-biome.

### Demonstrate microbiome dysbiosis

Diagnosing dysbiosis is of paramount importance as it can help anticipate the onset of various pathologies, given the association between the microbiome and its imbalances with diseases. The most challenging aspect of diagnosing dysbiosis lies in the individual variations of the microbiome and the interindividual variations that complicate the standardisation of results across different studies. Currently, there is no gold standard for diagnosing dysbiosis^133^; however, clinical evaluation remains the most effective. Gastrointestinal symptoms such as bloating, gas, diarrhoea, constipation, and abdominal discomfort can guide clinicians, but other nonspecific symptoms like fatigue, mood disturbances, and skin issues can also be associated with dysbiosis.^134^ Therefore, a comprehensive medical history, including details on antibiotic use, dietary habits, stress levels, and other factors influencing the gut microbiota, is crucial. As supplementary examinations, both stool analysis and breath tests can be conducted to assess inflammatory markers in the blood, providing additional guidance to clinicians.^135^ Additionally, metagenomics analysis aids in identifying the genomes of microbes, particularly the relatively small genome size of viral, bacterial, and many fungal members of the human microbiota.^136^ This analysis allows for the determination of their entire genetic makeup, especially for the more abundant members of a community. While there is no established gold standard, current microbiota analysis often relies on 16S rRNA gene amplification techniques.^133^ Overall, a multifaceted approach involving clinical evaluation, symptom assessment, and various diagnostic tests is essential for effectively demonstrating dysbiosis and guiding appropriate interventions.

### Prebiotics, probiotics, postbiotics and symbiotics

Prebiotics refer to selectively fermented components that induce specific alterations in the composition or activity of the gastrointestinal microbiota, thereby providing advantages to host health.^137^ The term “probiotic” refers to living non-pathogenic microorganisms administered in sufficient amounts to enhance microbial balance, particularly in the gastrointestinal tract.^138^ In 2021, the International Scientific Association for Probiotics and Prebiotics (ISAPP) introduced the term “postbiotic” as “a preparation of inanimate microorganisms and/or their components providing a health benefit to the host”.^139^ The synergistic combination of pro- and prebiotics, is a symbiotic.^140^

A variety of these treatments have been evaluated for restoring the intestinal microbiome. Prebiotics (fructo-oligosaccharide supplements, galacto-oligosaccharides, inulin, lactulose, breast milk oligosaccharides), probiotics (*Lactobacilli*, *Bifidobacteria*, *Enterococci*, *Saccharomyces boulardi*, *propionibacteria*, *Bacillus spp, E. coli* strain Nissle 1917) and symbiotics have been evaluated in chronic inflammatory bowel disease and psoriasis, with good results.^16,141^ For example, *Lactobacillus sp* and *Bacillus coagulans* probiotics have shown improved activity in patients with rheumatoid arthritis and spondyloarthritis.^142^ For oral microbiome, prebiotics (arginine, nitrate, β-methyl-D-galactoside and N-acetyl-D-mannosamine) and probiotics (*Lactococcus spp*) have been used to reverse oral microbiome dysbiosis with promising results. Nanotechnology may be use as drug delivery system, but clinical data remain limited to date.^143^ For respiratory microbiome, probiotics such as *Lactobacillus* administered orally or nasally, as well as prebiotics and symbiotics, have been used with promising results in limiting the prevention of respiratory infections.^144^ For vaginal and skin microbiomes, therapies to restore dysbiosis are fairly recent and not yet well established. Probiotics, prebiotics and symbiotics have been tested with promising results but still remain experimental.^145,146^

### Microbiota transplantation

Microbiota transplantation is the exogenous introduction of one or more micro-organisms into an individual with the aim of modifying his or her microbiome. It can be intestinal (faecal transplantation), cutaneous, vaginal, or even respiratory. It can be performed using a single microorganism or a microbiota cocktail. It has been most widely used for the intestinal microbiome. Faecal microbiome transplantation entails administering a solution of faecal matter from a donor into the recipient’s intestinal tract, directly shaping the recipient’s microbial composition for therapeutic benefits.^147^ It has mainly been evaluated using a Clostridium difficile ecosystem, but also with a transplantation of a bacterial consortium in chronic inflammatory bowel disease, obesity and other metabolic diseases, with encouraging results.^16^

The respiratory microbiome has an intrinsic relationship with the intestinal microbiome, which explains why faecal transplantation may also be beneficial at respiratory level.^144^ Some promising work has been carried out on the skin and vaginal microbiomes, but these are still at the experimental stage.^148,149^

### Antimicrobial therapies

Several antimicrobial strategies have been evaluated to restore dysbiosis. This includes: phage therapy, antimicrobial agents and peptides, predatory bacteria, biofilm-altering agents, acidifying agents, and photobiomodulation. Phage therapy involves using bacterial viruses (phages) to target harmful bacteria with precision.^150^ Bacteriophages and the use of predatory bacteria remain little-explored strategies for restoring the microbiome.^16^ The use of antimicrobial peptides (bacteriocins) and antimicrobial agents have shown promising results for oral microbiome dysbiosis.^86^ For the vaginal microbiome: photobiomodulation, which involves using red or near-infrared light in the vagina, the use of antimicrobials (antibiotics, antiseptics, antifungals), activated charcoal, biofilm-altering agents, acidifying agents, and phage therapy have been tested with good results but still remain experimental.^145,146^

### DMARDs, other immunomodulating or immunosuppressive drugs and microbiome

The use of DMARDs can affect the constitution of the microbiome and thus the dysbiosis. The most studied DMARDs are methotrexate, sulfasalazine and Etanercept in rheumatoid arthritis in human and animal models. But others csDMARDs and biologics like interleukin 1 or 6 inhibitors and other TNF alpha inhibitors may have beneficial actions especially on gout microbiome.^80^ Methotrexate has been shown to promote the growth of *Faecalibacterium*, and reduce that of *Enterobacteria* and *Haemophilus spp*, with variable action on *Bacterioides*. Sulfasalazine, on the other hand, is said to have an action on *azoreductase* bacteria, leading to an increase in *Bacillus* populations, and a decrease in *Bacteroidetes*, *E. coli,* and *Clostridium perfinges* colonies. Etanercept is thought to promote the proliferation of *Lactobacillus spp*, *Enterococcus* and *Cyanobacteria*, and reduce that of *Clostridiaceae* and *Bifidobacterium.*^151^ Methotrexate is the drug with the most evidence of beneficial action on the oral and intestinal microbiome.^152,153^ Certain non-conventional treatments with immunosuppressive and/or immunomodulatory effects have shown potential benefit in restoring microbiome balance, particularly in rheumatoid arthritis. These include traditional Chinese therapeutics such as Qingluo Tongbi Decoction and Triptolide, which have moreover been shown in animal models to have immunological regulatory effects on cells and pro- and anti-inflammatory cytokines.^154,155^

## CONCLUSION

The role of the microbiome in chronic inflammatory rheumatic diseases has become undeniable. It is consequently a key factor in therapeutic strategies for patients with chronic inflammatory rheumatic diseases. These strategies must necessarily be adapted to the population concerned and validated. However, there are still many uncertainties, and a great deal remains to be understood and known about the microbiome in patients suffering from chronic inflammatory rheumatic diseases prompting more research to be done.

## ABBREVIATIONS

AS: Ankylosing spondylitis;
CIRD: Chronic inflammatory rheumatic diseases;
DC: Dendritic cells;
DMARDs: Disease Modifying Anti-Rheumatic Drug;
RA: Rheumatoid arthritis;
SLE: Systemic lupus erythematosus;
SSc: Systemic sclerosis;
TGF beta: Tissue growth factor beta;
TNF alpha: Tumour necrosis factor alpha
